# Anchoring a Leviathan: How the Nuclear Membrane Tethers the Genome

**DOI:** 10.3389/fgene.2016.00082

**Published:** 2016-05-06

**Authors:** Rafal Czapiewski, Michael I. Robson, Eric C. Schirmer

**Affiliations:** The Wellcome Trust Centre for Cell Biology and Institute of Cell Biology, University of EdinburghEdinburgh, UK

**Keywords:** nuclear envelope, NET, gene position, NE-chromatin interaction, peripheral heterochromatin

## Abstract

It is well established that the nuclear envelope has many distinct direct connections to chromatin that contribute to genome organization. The functional consequences of genome organization on gene regulation are less clear. Even less understood is how interactions of lamins and nuclear envelope transmembrane proteins (NETs) with chromatin can produce anchoring tethers that can withstand the physical forces of and on the genome. Chromosomes are the largest molecules in the cell, making megadalton protein structures like the nuclear pore complexes and ribosomes seem small by comparison. Thus to withstand strong forces from chromosome dynamics an anchoring tether is likely to be much more complex than a single protein-protein or protein-DNA interaction. Here we will briefly review known NE-genome interactions that likely contribute to spatial genome organization, postulate in the context of experimental data how these anchoring tethers contribute to gene regulation, and posit several hypotheses for the physical nature of these tethers that need to be investigated experimentally. Significantly, disruption of these anchoring tethers and the subsequent consequences for gene regulation could explain how mutations in nuclear envelope proteins cause diseases ranging from muscular dystrophy to lipodystrophy to premature aging progeroid syndromes. The two favored hypotheses for nuclear envelope protein involvement in disease are (1) weakening nuclear and cellular mechanical stability, and (2) disrupting genome organization and gene regulation. Considerable experimental support has been obtained for both. The integration of both mechanical and gene expression defects in the disruption of anchoring tethers could provide a unifying hypothesis consistent with both.

## Introduction

It has been 130 years since Rabl (1885) used the ability to readily visualize chromosomes in salamander larvae to observe that genome organization is not random, noting that centromeres were located at the nuclear periphery. It took nearly 100 years before this seminal discovery was finally tested by Cremer et al. (1982), who in the years following developed many tools and ideas to advance our understanding of the nature of chromosome territories and their positioning in the nucleus. At roughly the same time biochemical evidence for a physical interaction of chromatin with the nuclear envelope (NE) was supported by NE retention of chromatin after extraction at high ionic strengths (Bouvier et al., 1985). Since then, it has been shown that chromosomes and specific genes tend to have preferred radial positions in the nucleus with respect to the nuclear periphery (Croft et al., 1999), that some of this spatial organization is tissue-specific (Kim et al., 2004; Parada et al., 2004), and moreover that some specific genes actively reposition between the nuclear interior and the nuclear periphery and their position correlates with their activation state (Kosak et al., 2002; Hewitt et al., 2004; Zink et al., 2004; Williams et al., 2006). Separately heterochromatin, both as originally defined by dense negative staining material in electron micrographs (Mirsky and Allfrey, 1960; Hirschhorn et al., 1971) and according to the modern definition of epigenetic silencing marks (Minc et al., 1999; Pickersgill et al., 2006; Pindyurin et al., 2007), was found concentrated at the nuclear periphery.

**FIGURE 1:**
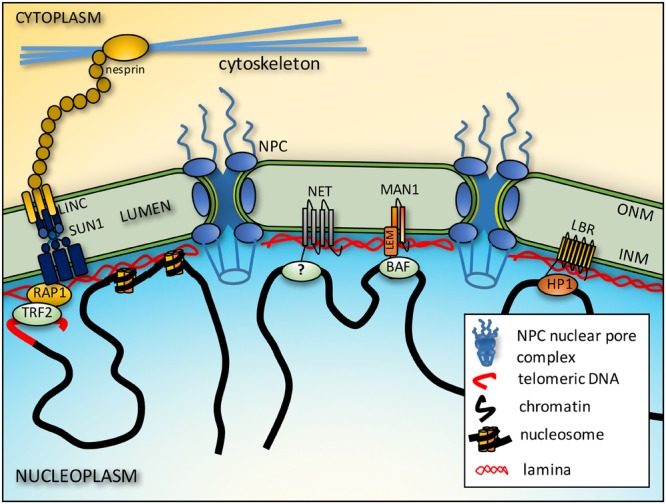
**Connections between chromatin and the NE.** Telomeres interact with the NE from interactions of proteins on the telomeres such as RAP1 and proteins embedded in the inner nuclear membrane (INM) such as SUN1. These interactions in turn may be stabilized by further connections across the lumen of the NE to outer nuclear membrane (ONM) nesprins that in turn connect to cytoplasmic filaments. The intermediate filament lamin polymer that lines the INM interacts with core histones to also tether chromatin to the NE. There are also more general interactions between LEM domain (a domain shared by LAP2β, emerin and MAN1) NETs and the chromatin protein barrier-to-autointegration factor (BAF), but some NETs appear to have more tissue-specific interactions though the chromatin partners responsible have yet to be identified (?). Finally, some NETs preferentially bind heterochromatin proteins such as LBR binding heterochromatin protein 1 (HP1).

The NE (**Figure 1**) is the defining structure at the nuclear periphery. It is a double membrane system rich with NE transmembrane proteins (NETs), many of which are tissue-specific, and stabilized by its own structural protein network, the nuclear lamina (Worman and Schirmer, 2015). The outer nuclear membrane (ONM) is continuous with the endoplasmic reticulum and contains several NETs that connect to the three major cytoplasmic filament systems. Several of these are from a family of KASH domain proteins called nesprin/syne proteins (Luxton and Starr, 2014). The ONM connects to the inner nuclear membrane (INM) at sites where megadalton nuclear pore complexes (NPCs), that direct transport of proteins and RNA in and out of the nucleus, are inserted (Dickmanns et al., 2015). The INM contains hundreds of NETs of which the majority tested bind chromatin proteins and/or lamins (Kind and van Steensel, 2014; Wong et al., 2014; Harr et al., 2016). Lamins form a polymer lining the inner surface of the INM and also interact directly with the genome (Gruenbaum and Foisner, 2015). The lamin polymer and NETs it interacts with together make the structural protein network called the nuclear lamina. The lamin polymer also interacts indirectly with cytoplasmic filaments through interactions with INM SUN domain NETs that in turn connect with the ONM KASH domain NETs in what is referred to as the linker of nucleo- and cytoskeleton (LINC) complex (Padmakumar et al., 2005; Crisp et al., 2006). Lamins and LINC complex proteins mediate force transmission between the nucleus and cytoplasm (Lombardi et al., 2011) and accordingly are important for overall nuclear mechanical stability, cell and nuclear migration, and mechanosignal transduction (Ho et al., 2013; Swift et al., 2013). The NE lumen is mostly unexplored territory, but some luminal proteins such as Torsin A are known to interact with INM NETs (Goodchild and Dauer, 2005).

This incredibly complex structure disassembles and reassembles in each mitosis of higher organisms (Schellhaus et al., 2015). On the one hand this dynamic behavior could be viewed as an obstacle to maintaining spatial genome organization, but it is also an opportunity for a differentiating or activated cell to rapidly change its genome organization pattern. In support of the latter, reversal of artificially induced positioning changes required mitosis (Finlan et al., 2008; Reddy et al., 2008) and reversal of NET-induced genome organization patterns upon NET knockdown became stronger with increasing cell divisions (Zuleger et al., 2013). Separate, rapid chromosome movements within 15 min of senescence induction depend on motor proteins (Bridger et al., 2000; Mehta et al., 2010) and there are also more protracted larger scale changes in post-mitotic cells that take weeks (Solovei et al., 2013); so there are clearly multiple mechanisms involved in establishment of spatial genome organization with respect to the NE.

The functional relevance of these interactions and patterns of spatial genome organization is not yet fully clear; however, they are evidently important because such organization is visibly disrupted in several diseases linked to mutations in NE proteins. Specifically by electron microscopy dense chromatin that is normally directly apposed to the NE redistributes away from the membrane or is reduced at the periphery in patients with both NET (emerin) and lamin-related muscular dystrophy (Fidzianska et al., 1998; Ognibene et al., 1999; Sewry et al., 2001; Maraldi et al., 2002; Verga et al., 2003), progeria (Goldman et al., 2004), mandibuloacral dysplasia (Maraldi et al., 2006) and familial partial lipodystrophy, Dunnigan-type (Maraldi et al., 2006) and nuclear lobulated structure associated with chromatin in neutrophils is altered in NET (LBR)-related Pelger–Huet anomaly (Hoffmann et al., 2002). Some differences in the overall positioning of chromosomes were also observed in cells with specific lamin A mutations (Meaburn et al., 2007; Mewborn et al., 2010). In the case of Hutchison-Gilford Progeria Syndrome, cells were also stained for epigenetic marks and the loss of dense peripheral chromatin correlated with the loss of epigenetic silencing H3K9 and H3K27 tri-methylation marks (Shumaker et al., 2006). Important questions before us are: how are these patterns established and maintained? How does spatial genome organization contribute to genome regulation? Do disease pathologies reflect disruption of specific critical genes or of random collections of genes altered by global mechanical disruption of NE-genome connections?

## Genome Interactions at the NE

The peripheral localization of centromeres discovered by Rabl (1885) is not typical in mammalian cells, though the recruitment of centromeres to the periphery has been observed in granulocyte differentiation into neutrophils (Aquiles Sanchez et al., 1997) and in myogenesis (Chaly and Munro, 1996; Rozwadowska et al., 2013). In both cases nothing is known about how the recruitment and tethering works.

### Telomeres

Much more is known about the conserved tethering of telomeres to the periphery. From yeast to man, telomeres associate at least transiently with the NE through interactions involving SUN domain NETs. However, the first studies investigating the actual proteins involved in the yeast *Saccharomyces cerevisiae* identified the Ku proteins on the telomeres themselves (Laroche et al., 1998). Subsequently the NPC protein TPR was also implicated (Galy et al., 2000) in the anchoring tether. Several years later, studies in the fission yeast *Schizosaccharomyces pombe* revealed the involvement of Sad1, a SUN domain NET (Chikashige et al., 2006). Shortly afterward, SUN proteins were found to be as important as the other players in *S. cerevisiae* (Antoniacci et al., 2007; Bupp et al., 2007) and mammalian spermatocyte meiosis, though one study implicated SUN1 (Ding et al., 2007) while another implicated SUN2 (Schmitt et al., 2007). These proteins appear to have partially redundant and partially distinct functions. There are likely to be differences in telomere tethering complexes because organisms like budding yeast maintain telomeres at the NE while mammals principally anchor telomeres transiently in meiosis, where they facilitate chromosome alignment in synaptonemal complex formation and thus genetic crossover events (Scherthan et al., 1996). In addition to the more specialized meiosis, SUN-telomere interactions occur in post-mitotic NE reassembly that involves both SUN1 and the shelterin subunit RAP1 (**Figure 1**) (Crabbe et al., 2012). Lamins may be involved in both telomere and centromere tethering because a mutation causing Hutchison-Gilford progeria syndrome, E145K, yielded an abnormal distribution of telomeres and clustering of centromeres (Taimen et al., 2009).

### General Chromosomal DNA

Several reports demonstrated that lamins bind DNA (Luderus et al., 1992, 1994; Rzepecki et al., 1998); however, cytoplasmic intermediate filaments could bind DNA *in vitro* (Shoeman and Traub, 1990), suggesting that this interaction might be a non-specific characteristic of the conserved intermediate filament rod. This view was strengthened by separate studies showing that lamins bind core histones with higher affinity, specifically H2A and H2B (**Figure 1**) (Hoger et al., 1991; Taniura et al., 1995; Goldberg et al., 1999). With an estimated 9 million copies of lamins per mammalian cell nucleus (Schwanhausser et al., 2013) and multiple binding sites lamins should be major contributors to the aggregate chromatin-NE interaction. Nonetheless, as these interactions are quite general it is hard to imagine how they could contribute specificity to spatial genome organization. It is possible that different lamin subtypes — lamins are encoded by three genes, each of which has multiple splice variants — have distinct and higher affinities for particular histone variants, which could confer some specificity. This possibility has yet to be investigated and should be tested.

The NETs LAP2β and MAN1 can both directly bind DNA (Cai et al., 2001; Caputo et al., 2006); however, the majority of NET interactions identified have been with chromatin proteins or chromatin-associated proteins. LAP2β also, like lamins, can bind core histones (Foisner and Gerace, 1993), but it can also bind the chromatin protein barrier-to-autointegration factor (BAF) (Furukawa, 1999). BAF can directly bind both DNA and histones and so is thought to promote greater chromatin compaction (Zheng et al., 2000; Skoko et al., 2009). NETs emerin and MAN1 also bind BAF (Lee et al., 2001; Mansharamani and Wilson, 2005).

### Boundary Elements

Early investigations of NPC-chromatin interactions yielded conflicting results on gene regulation with evidence of both activating and silencing roles. However, some of this was clarified in yeast with the discovery of boundary elements where NPC connections segregate active and silent regions (Ishii et al., 2002). Mammalian NPCs appear to be different where some NPC components have two separate pools: one activates genes in the nucleoplasm while the other in the peripheral NPC structures has a silencing function (Capelson et al., 2010; Kalverda et al., 2010). Experiments fusing a soluble NPC protein to a heterologous transmembrane domain nicely demonstrated that the nuclear periphery per se has unique properties for gene regulation.

### Heterochromatin

Most heterochromatin tends to be located either at the nuclear periphery both visually by electron microscopy and as assessed by genome-wide targeted sequencing of DNA proximal to the lamina (Pickersgill et al., 2006). This more modern approach fuses lamin B1 to a bacterial dam methylase to label peripheral DNA with a unique type of methylation that allows its enrichment for sequencing and refers to lamina-associated domains as LADs. Most LADs are conserved in different cell types and so called “constitutive” while a subset of “facultative” LADs change during differentiation (Peric-Hupkes et al., 2010). Much more dramatic than these normal changes in differentiation is the complete inversion of peripheral heterochromatin and euchromatin in the nuclei of rod photoreceptors in nocturnal mammals (Solovei et al., 2009). This complete radial reorganization of heterochromatin depends interchangeably on lamin A or the NET LBR (Solovei et al., 2013). At the same time, though LADs are identified by lamin interactions, LAD organization was mostly unaffected with lamin knockout, indicating that additional factors likely contribute (Amendola and van Steensel, 2015).

LBR binds heterochromatin through a direct interaction with HP1α and HP1γ (**Figure 1**) (Ye and Worman, 1996) and global analysis of chromatin that coimmunoprecipitated with LBR revealed a strong enrichment in silencing epigenetic marks (Makatsori et al., 2004). Moreover, the accumulation of microinjected HP1α at the periphery before eventually being distributed to other nuclear locations (Kourmouli et al., 2000) argues for a higher affinity of binding at the periphery than internal locations, though this has never been tested. Interestingly, LBR is also reported to bind MeCP2 that binds to methylated DNA (Guarda et al., 2009).

An excellent and detailed review of all epigenetic chromatin marks found at the nuclear periphery can be found in (Harr et al., 2016). In support of the importance of epigenetic silencing marks for peripheral genome organization, siRNA depletion or pharmacological disruption of enzymes that deposit these silencing marks and their readers disrupted peripheral gene positioning (Kind et al., 2013; Gonzalez-Sandoval et al., 2015). These interactions can also be important for tissue-specific genome changes in development as depletion of the H3K9 methyl binding chromodomain protein CEC-4 inhibited myogenesis (Gonzalez-Sandoval et al., 2015).

### Chromosome and Gene Positioning

Gene-poor chromosomes tend to be at the nuclear periphery while gene-rich chromosomes tend to be internal (Croft et al., 1999; Boyle et al., 2001; Bolzer et al., 2005; Wiblin et al., 2005; Guelen et al., 2008). This radial organization can be modulated for specific chromosomes by the physiological state of the cell, for example senescence (Bridger et al., 2000; Mehta et al., 2010). Moreover, a subset of chromosomes changes their radial nuclear position in specific tissues. For example mouse chromosome 5 tends to be in the nuclear interior in liver while being at the periphery in lung (Parada et al., 2004) and chromosome 6 is in the interior in CD4+ T-cells and at the periphery in CD8+ T-cells (Kim et al., 2004).

There are likely to be many players in all these types of chromosome positioning that generate distinct anchoring tethers, but it is clear that both lamins and NETs are important. Chromosome 18 peripheral localization reflects the gene density distribution and this chromosome moves away from the periphery in lamin B1 knockout cells with resultant gene de-repression (Malhas et al., 2007), indicating the functional relevance of this positioning. It seems likely that heterochromatin interactions also contribute. Lamin A and emerin are likely important for positioning affected by physiological changes because mutations in these two proteins linked to disease perturb the normal positioning patterns for chromosomes that normally reposition upon serum withdrawal (Meaburn et al., 2007; Mehta et al., 2011). Finally, tissue-specific NETs are important for at least some tissue-specific patterns of radial chromosome positioning (Korfali et al., 2010; Zuleger et al., 2013). However, the relevance of overall chromosome positioning to disease is questionable as two *LMNA* mutations causing cardiomyopathy had different effects: E161K results in loss of chromosome 13 from the periphery while D596N maintains chromosome 13 at the periphery (Mewborn et al., 2010). As only whole chromosome movements were tested in this study it remains possible that individual gene repositioning could contribute to disease.

Pathologies could occur if critical genes within chromosomes lose normal positioning with consequent effects on their regulation. Combinatorial FISH for individual genes and the chromosomes they are on revealed that many genes reposition during differentiation without their host chromosome also repositioning (Morey et al., 2008; Szczerbal et al., 2009), suggesting that bulk chromosome movements may reflect just an aggregate of individual gene relocalizations. Many important genes have been found to reposition radially concordant with changes in their expression (**Figure 1**). For example, the immunoglobulin heavy chain *IgH* locus is at the nuclear periphery in early lymphocyte lineages but moves to the nuclear interior roughly when V(D)J recombination is initiated (Kosak et al., 2002). In neurogenesis *Mash1* (*Ascl1*) moves away from the periphery concomitant with its activation (Williams et al., 2006). Many other examples of genes under such regulation have been described from the *FABP4* gene important for adipogenesis (Szczerbal et al., 2009) to transcription factor loci (Hewitt et al., 2004) to the cystic fibrosis transmembrane conductance regulator (CFTR) gene (Zink et al., 2004).

In most cases the molecular nature of the tether and how it is regulated is unknown. However, a mechanism was indicated in *S. cerevisiae* where movement of the *INO1* gene to the periphery upon transcriptional inactivation required replacement of local histones with the histone variant H2A.Z and the NET Scs2p (Brickner and Walter, 2004; Brickner et al., 2007). Thus, a combination of a unique chromatin mark on a gene together with a NET that presumably has an affinity for this particular mark could confer specificity to anchoring tether interactions.

## Postulated Gene Regulatory Mechanisms From NE-Directed Genome Organization

NE-genome interactions clearly add an additional layer to gene regulation, but resolving specific effects and mechanisms has proven difficult. The case of telomeres is a good example. Inserting genes close to yeast telomeres resulted in their silencing in a process involving Sir Proteins (Gottschling et al., 1990; Aparicio et al., 1991). At first it was thought that this silencing was purely a unique function of telomeres, but several years later both the Sir silencing proteins and telomeres were found to be concentrated at the periphery (Gotta and Gasser, 1996; Maillet et al., 1996). The identification of Ku proteins on telomeres and Mlp proteins of the NPC as players in the peripheral localization of telomeres (Laroche et al., 1998; Galy et al., 2000) enabled targeted disruption of the telomere peripheral association and testing the effect on the silencing. Mutations in these proteins yielded de-repression of silenced genes in yeast (Galy et al., 2000; Maillet et al., 2001; Feuerbach et al., 2002) and the same effect was observed for knocking down the mammalian Mlp homolog Tpr (Scherthan et al., 2000).

In the opposite direction, tethering a reporter gene to a nuclear membrane protein resulted in silencing of the reporter (Andrulis et al., 1998). While this was first thought to represent an NPC function, it was later found that some Sir proteins interact with NETs in areas distinct from telomeres (Andrulis et al., 2002), indicating that silencing is a general property of the periphery. As the functions of Sir proteins in epigenetic silencing came to light it was postulated that changes to the state of chromatin when at the periphery were responsible for silencing as opposed to a requirement to be physically at the periphery. Using more complex systems to recruit and then release a reporter found that breaking the connection to the NE did not de-repress the silent reporter (Gartenberg et al., 2004). As each study used different artificial experimental systems, this question remains unanswered in yeast and it may differ in mammalian cells but from existing data arguments can be made for steric position effects, chromatin modifications from silencing enzymes, physically separating chromosome regions, and other unique properties of the periphery.

### Steric Factors

**FIGURE 2:**
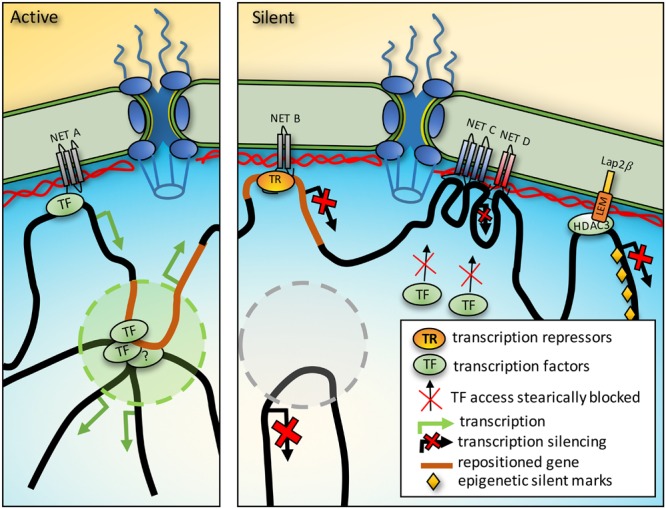
**Mechanisms of gene regulation from the nuclear periphery.** Activation. NET binding to transcription factors (TF) could promote activation of a gene also sequestered to the periphery. Furthermore, genes between chromatin anchoring points could be directed into TADs where for example the presence of a distal enhancer could strengthen expression. Silencing. In a different cell type the same region that was activating in a TAD could be recruited to the periphery to prevent the enhancer interaction and silence it. Just as TFs could be concentrated at the periphery by NET binding, so could transcriptional repressors (TR). Densities from protein complexes around genes tethered at the NE could also prevent access to TFs. Finally, some NETs interact with enzymes that add epigenetic marks and so recruitment of a gene to the periphery could promote the acquisition of silencing marks.

A transcriptional regulators ability to physically access binding sites on DNA could be blocked by large complexes that prevent access or if the binding sites are already occupied by a higher affinity partner (**Figure 2**). This is consistent with observations that transgenes located near the nuclear periphery in mammalian cells are less mobile than those residing in more internal positions (Chubb et al., 2002). Similarly, a genome-wide study of plasmid integration found that internal sites are greatly favored for integration over peripheral sites (Akhtar et al., 2013) and natural viral integration similarly avoids the periphery (Marini et al., 2015). At the same time, soluble molecules can travel quickly through such environments (Grunwald et al., 2008), so further technological advances in imaging or indirect biophysical approaches will be needed to clarify this possibility.

### Adoption of Epigenetic Marks Once at the Periphery

When at the periphery the *Mash1* locus had histone modifications characteristic of silenced chromatin, but after it moved to the interior to become activated in neural-committed cells the histone modifications were consistent with active chromatin (Williams et al., 2006). However, as this process required induction of differentiation with a panoply of changes in transcription factors, transcriptional repressors and epigenetic marks throughout the genome it was impossible to determine whether these epigenetic changes preceded or followed the locus repositioning.

The ability to manipulate the peripheral association of an artificial locus enabled testing the effects of changing position without extraneous effects of differentiation. Three groups generated an artificial locus using bacterial LacO sequences and found the locus was in the interior in cells expressing GFP fused to the LacI repressor that binds LacO sequences while the locus moved to the periphery if the LacI was fused to a NE protein (Finlan et al., 2008; Kumaran and Spector, 2008; Reddy et al., 2008). Though the studies differed in the effects of peripheral re-localization on gene regulation and the acquisition of silencing marks, in two studies histone H4 acetylation at the artificial locus was reduced when the locus was at the periphery (Finlan et al., 2008; Reddy et al., 2008) consistent with the periphery containing enzymes that add epigenetic silencing marks.

One such enzyme is histone deacetylase 3 (HDAC3) that removes H4 acetylation and binds the NETs LAP2β and emerin (Somech et al., 2005; Demmerle et al., 2012). Thus the NET-HDAC3 interaction enables silencing of genes that come into contact with the periphery (**Figure 2**). In the other direction, targeting a protein that unfolds DNA, presumably removing silencing marks, was sufficient to move an endogenous locus away from the periphery suggesting these interactions help maintain genes at the NE (Therizols et al., 2014).

### Localized Concentration of Transcriptional Regulators

NET binding to transcriptional repressors sitting on particular genes could recruit those genes to the NE and increase the localized transcriptional regulator concentration at the periphery to promote further repression (**Figure 2**). If the volume of the nuclear periphery is considered as 50 nm in from the membrane it would account for roughly 1/40th of nuclear volume in a typical mammalian cell. Thus, NET-transcriptional regulator binding would be the equivalent of increasing its expression 40-fold for a target also located at the periphery. The transcriptional repressors germ cell-less and Btf bind emerin and LAP2β (Nili et al., 2001; Holaska et al., 2003; Haraguchi et al., 2004) and this interaction is functional because germ cell-less mediates specific repression of E2F-regulated genes when LAP2β is overexpressed (Nili et al., 2001; Holaska et al., 2003).

### Topologically Associated Domains (TADs)

The genome is organized into local topologically associated domains (TADs) that can further interact in higher-level chromatin compartments so that genome regions tens of megabases away from one another can also interact (Dekker et al., 2013). The exact structure of the chromatin in TADs is not known, but chromatin loops stabilize TADs (Giorgetti et al., 2014) and chromatin looping is known to contribute to cooperative chromatin functions (Griffith et al., 1986). Thus two genome connections at the NE could produce a chromatin loop between them that could participate in the internal organization of the nucleus, for example by positioning an enhancer proximal to a target gene that is hundreds of kilobases distant, and recruiting this enhancer to the NE could prevent its functioning with its target gene (**Figure 2**). Such NE-genome interactions could help explain how TADs are assembled which is currently a mystery.

Some TADs are clearly functionally important as deletions/inversions around a developmental locus (*WNT6/IHH/EPHA4/PAX3*) disrupted TAD structure with corresponding developmental defects (Lupianez et al., 2015). The CTCF protein is often found associated at TAD boundaries and another study found that disease-linked SNPs at such CTCF-binding sites disrupted the TADs (Guo et al., 2015), suggesting that the disease state is caused by loss of genome organization with corresponding gene misregulation.

### Gene Activation at the NE

Although the majority of observations reflect gene repression at the NE, genes can also be activated at the NE. For example, the proteolipid protein (PLP) gene undergoes the transition from inactive to active while remaining at the periphery during oligodendrocyte differentiation (Nielsen et al., 2002). Global analysis also indicates that a subset of genes that move to the periphery in differentiation are activated (Peric-Hupkes et al., 2010). As several NETs bind transcription factors such as Lmo7 and Smads (Osada et al., 2003; Pan et al., 2005; Holaska et al., 2006), gene activation could function similarly to the localized concentrations of transcriptional repressors noted above.

Finally, just as the epigenetic silencing enzyme HDAC3 was found to associate with a NET, the epigenetic activating enzyme hALP (also called NET43 and NAT10) interacts with the NET SUN1 (Chi et al., 2007). This interaction appears to promote mitotic chromosome decondensation at the end of mitosis as depletion of SUN1 results in delayed decondensation and a reduction in histone H2B and H4 acetylation in a hALP-dependent manner (Chi et al., 2007). Another reflection of the potential importance of such NE-genome interactions to human disease is the ability of treatment with a hALP inhibitor to reverse defects in Hutchison-Gilford Progeria Syndrome cells in tissue culture (Larrieu et al., 2014).

## Mechanisms for Tethering Genes to the NE

A gene is not localized at the NE in isolation, but remains part of a gigadalton chromosome that may exert considerable force on the gene-tethering NET in the membrane. Therefore, NETs likely bind other NE proteins to stabilize the gene tether.

### Affinity Tethering

The LacO-LacI system mentioned earlier operates by the very high affinity of LacI to bind LacO sequences. Thus these experiments demonstrated the ability to reposition a locus to the NE based on affinity tethering (Finlan et al., 2008; Kumaran and Spector, 2008; Reddy et al., 2008). Moreover, one study used lamin B1-LacI fusions (Kumaran and Spector, 2008) while the others used different NET-LacI fusions (Finlan et al., 2008; Reddy et al., 2008), indicating that both lamins and NETs can function as NE tethers.

These studies used 100s of copies of the LacO array in their artificial locus and endogenous genes are not likely to have so many proximal binding sites that could participate in a tether. This raises the hypothesis that either multiple proximal interactions help to stabilize a particular anchor and to stabilize an individual anchoring tether will require many proteins functioning together in a complex both on the chromatin side and on the NE side.

### Multi-Protein Complexes

As the telomere example was the first characterized molecularly and is the most studied, it is also the example where the most players have been identified. In *S. pombe* the SUN protein was found to also work with two new proteins Bqt1 and 2 (Chikashige et al., 2006). In *S. cerevisiae* the SUN protein worked together with the Ctf7p cohesion factor and Est1p telomere associated protein (Antoniacci et al., 2007). The mammalian meiotic telomere tether further includes the full LINC complex with gamete-specific KASH5 contributing the nesprin part of the complex (Morimoto et al., 2012). Other studies found additional complex components such as CCDC79/TERB1 on the telomeres and the meiosis specific cohesin SMC1B (Daniel et al., 2014). Complex complexity is increased by observations that telomere-NE tethering is regulated through phosphorylation by CDK2 (Viera et al., 2015). Finally, there is likely to be a different complex in mitosis where rapid movements of telomeres in prophase require SUN1, KASH5, dynein and microtubules (Lee et al., 2015).

Multi-protein complexes clearly are also important for specific gene tethering. The NET LAP2β together with its silencing partner HDAC3 and the transcriptional regulator cKrox were all required for NE tethering of the *IgH* and *Cyp3*a loci (Zullo et al., 2012). However, this was tested in fibroblasts and these are loci that are developmentally regulated in lymphocytes; so there are probably additional regulatory and tissue-specific components of the complex such as the tissue-specific NETs involved in chromosome positioning (Korfali et al., 2010; Zuleger et al., 2013). Another complex including at least emerin and HDAC3 appears to be important for the positioning of Myf5, MyoD, and Pax7 myogenic genes (Demmerle et al., 2013).

### Force Distribution Through the Lamina

**FIGURE 3:**
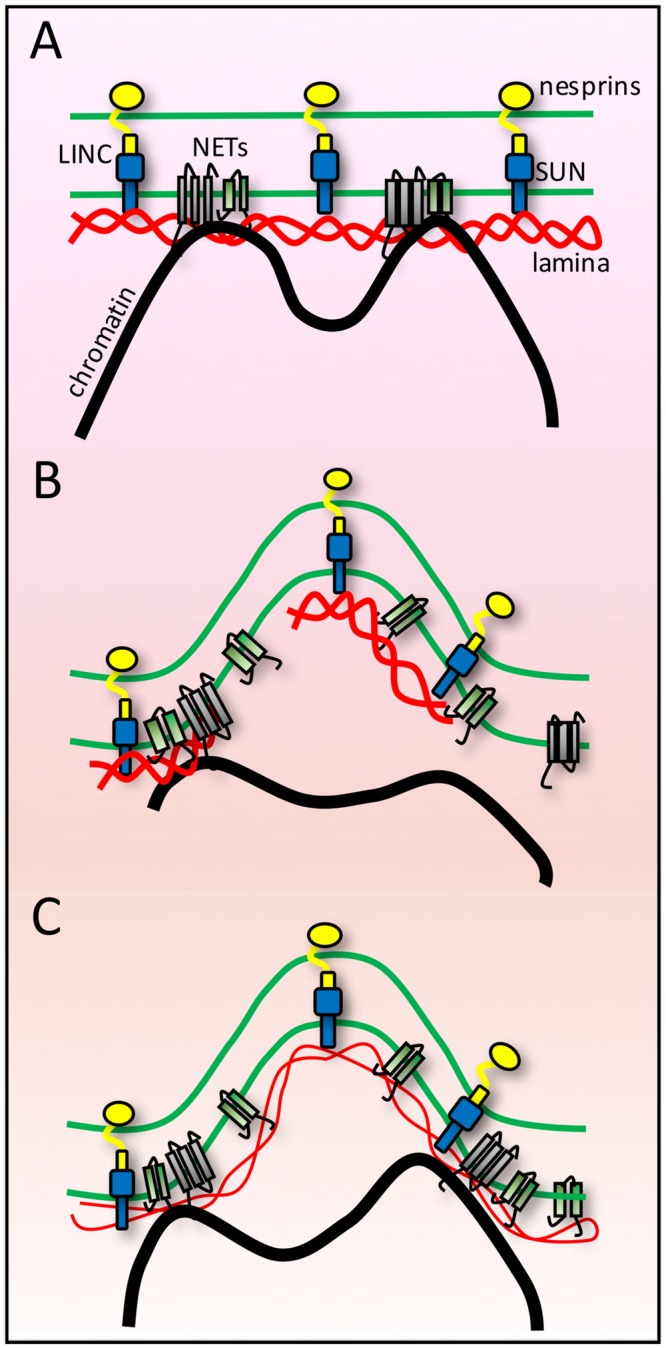
**Lamina buffering of forces from anchoring tethers. (A)** High-resolution microscopy on fixed cells often shows many nuclear invaginations while live cell imaging of shows that nuclei are subject to transient deformations. As the NE endures forces from both genome and cytoskeletal interactions these findings can be interpreted as indicating the NE is at the same time extremely sturdy and extremely flexible and that individual connections must be tightly anchored for such large visible structural changes to occur in response to such forces. **(B)** If the lamin polymer was less strong than the forces exerted upon it, then it might break under the forces. Likewise if the polymer was strong but chromatin was not strongly anchored, then the chromatin might break away from the NE. **(C)** A combination of strong tethering anchors and a strong but flexible lamin polymer would allow the nuclear structural changes observed.

To withstand the strong pushing and pulling biophysical forces from chromosomes a tethering anchor would likely require both considerable strength and elasticity. Such characteristics can be found in the nuclear lamina. Intermediate filaments maintain their integrity under strain and stretch forces that tear apart microtubules and actin filaments (Janmey et al., 1991). Thus it is probably no mistake that the NE contains ONLY intermediate filaments of these three primary cellular filaments, especially as the NE needs to withstand strong forces on both sides — from the genome and from cytoplasmic filament systems (**Figure 3**).

The binding of lamins to most NETs tested adds to the beauty of the system as it tightly links the membrane also to the lamin polymer, thus further distributing forces from NE-genome interactions. Furthermore, as the lamin polymer is one large network that lines the whole INM this means that ALL NE-genome interactions connect to the same protein network. Thus, chromosome forces from one tether point could be counterbalanced by opposing chromosome forces from another tether point on the same larger lamina network. The aggregate complexity of interactions should be able to keep chromatin tethered while still being able to stretch in response to forces placed on the lamin-NET-membrane network by genome movements.

What is most missing from this model is a structural understanding of the lamin polymer. Different lamin subtypes have distinct *in vitro* binding strengths and *in vivo* network stabilities (Schirmer and Gerace, 2004; Lammerding et al., 2006). The different subtypes also appear to assemble into largely distinct and layered polymers (Goldberg et al., 2008; Shimi et al., 2015). However, while cytoplasmic intermediate filaments can be clearly visualized by electron microscopy as 10 nm filaments, the nucleoskeletal lamins have only been visualized in oocytes from lower vertebrates such as *Xenopus laevis* because in this system chromatin is not in direct contact with the lamin polymer. Recent reports suggest that the diameter of lamin filaments may be variable (Goldberg et al., 2008; Shimi et al., 2015) and thus one of the most important issues for the field right now is to determine the actual structure of the lamin polymer.

### The Nut-and-Bolt Model

**FIGURE 4:**
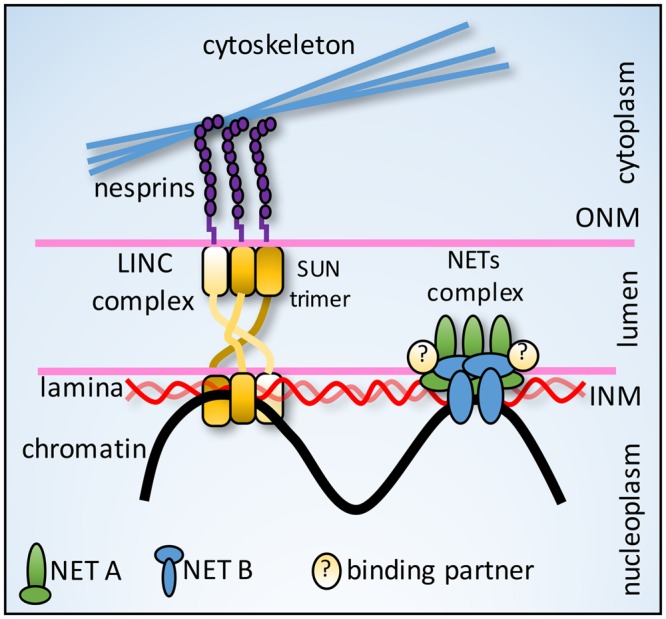
**Nut-and-bolt model for anchoring tethers.** Nesprins (KASH-domain NETs) in the ONM connect in the lumen just under the membrane to SUN protein NETs in a trimeric interface stabilized by a triple helix formed by the three SUN protein molecules. This fanning out under the membrane could better distribute forces to withstand pulling forces from cytoplasmic filaments. NE-genome anchoring tethers could similarly utilize interactions with other NETs and luminal proteins to distribute chromosomal forces and prevent the NETs from being pulled from the membrane.

The LINC complex that connects the nucleoskeleton to cytoplasmic filaments forms a triple helix interface just below the outer membrane (Sosa et al., 2012; Zhou et al., 2012), fanning out like a nut stabilizing a bolt (**Figure 4**). This structure could distribute force under the membrane to prevent cytoplasmic filaments from pulling LINC components out of the membrane. We hypothesize that NETs involved in anchoring tethers to the genome require a similar structure to counter forces from gigadalton chromosomes (**Figure 4**). Thus, anchoring NETs could interact with other NETs in the INM, particularly those with considerable luminal mass to distribute force on the other side of the membrane and they could also interact with completely luminal proteins. For example, the NETs LAP1 and NET9/LULL1 interact with torsin A in the lumen (Goodchild and Dauer, 2005): if these NETs also interacted with a NET directly involved in chromosome or gene positioning such as NET47/TM7SF2 (Zuleger et al., 2013), then the greater complex would similarly spread out in the lumen of the NE to distribute force from the NET-chromosome interaction. Also supporting this idea is that quantifying NET mass in the NE lumen based on topology prediction indicates that for most NETs a majority of their mass lies in the lumen (Kavanagh et al., 2007; Zuleger et al., 2011).

### The Dynamic Scaffold

Some individual loci in the nuclear interior have been observed to move rapidly over large distances during interphase, especially when they are becoming activated (Tsukamoto et al., 2000; Chuang et al., 2006). In contrast, loci at the nuclear periphery tend to be much less mobile (Chubb et al., 2002). Nonetheless, at least some peripheral loci dynamically exchange their tethers as recent advances in the DamID method to label peripheral DNA enabled live cell mapping that revealed some LADs change during interphase (Kind et al., 2013).

An anchoring tether involves both the DNA/chromatin and the NE proteins. Fluorescence recovery after photobleaching (FRAP) and photoactivation experiments on NETs involved in chromosome repositioning revealed that one population of these NETs is extremely dynamic while another population is not (Zuleger et al., 2011, 2013). For example, LAP2β t½ by FRAP was 25.0 s which was not much different from its photoactivation t½ for ER to NE translocation of 14.6 s. However, its photoactivation t½ for NE to NE movements was 70.2 s, arguing that there are at least two populations of high and low mobility. The question remains whether the low mobility population reflects a dynamic tether or if the much smaller (5–10% of total) immobile population that never recovers by FRAP reflects a more protracted tether.

## Conclusion

We predict that all the mechanisms described above contribute to overall gene positioning and regulation in complex organisms. Tissue-specific NETs likely function in complexes together with other NETs, the lamin polymer and luminal proteins that generate anchoring tethers that bind to specific proteins on genes requiring tighter regulation. For example, a gene from an alternative differentiation pathway that needs to be very strongly shut down or a gene that needs to be temporally regulated because it is needed early but becomes inhibitory to differentiation if expressed later. We postulate that tissue-specific gene tethering and heterochromatin tethering function synergistically, each facilitating the establishment of the other. For example, a high affinity interaction setting up a tissue-specific anchor is strengthened by the addition of epigenetic silencing marks once at the periphery and then more abundant heterochromatin-NE accumulate to stabilize the anchor. The cumulative interactions would then further stabilize chromosome territories. This is just a hypothesis at this stage and to test it the next stages of investigation need to focus on identifying all proteins in a tethering anchor, measuring their relative binding affinities for chromatin proteins, lamins and other NETs, and determining their dynamic behavior. Most importantly this work needs to proceed in tissue differentiation systems measuring endogenous proteins and loci to fully understand the molecular mechanisms and consequences for genome regulation and the role of these anchoring tethers in human disease.

## Author Contributions

ES wrote the manuscript with help from RC and MR. Ideas and hypotheses came from discussions between all authors. RC made figures and MR helped particularly with references.

## Conflict of Interest Statement

The authors declare that the research was conducted in the absence of any commercial or financial relationships that could be construed as a potential conflict of interest.
